# Contract Termination and Insurance Enrollment Among Medicare Advantage Beneficiaries

**DOI:** 10.1001/jamanetworkopen.2024.28267

**Published:** 2024-08-20

**Authors:** Meehir N. Dixit, Amal N. Trivedi, David J. Meyers

**Affiliations:** 1 Department of Health Services, Policy, and Practice, Brown University School of Public Health, Providence, Rhode Island

## Abstract

**Importance:**

Approximately one-fifth of Medicare Advantage (MA) contracts terminated their participation in the MA program between 2011 and 2020. Little is known about subsequent insurance choices following a termination.

**Objective:**

To examine the insurance destinations of MA enrollees and the characteristics of enrollees who switch into traditional Medicare (TM) after a contract termination.

**Design, Setting, and Participants:**

This cross-sectional study examined MA program data of MA beneficiaries in the Medicare Master Beneficiary File from 2016 to 2018. Statistical analysis was performed from June 2023 to April 2024.

**Exposures:**

Beneficiary characteristics, including age, sex, race and ethnicity, dual eligibility; hospital, nursing home, and home health utilization; and contract characteristics, including plan type, vertical integration, premium, and MA star rating.

**Main Outcome and Measures:**

The main outcome was switching to TM in the year immediately after termination. We also evaluated the characteristics of contracts among those who remained in MA.

**Results:**

A total of 117 681 beneficiaries were included in this analysis (64 654 [54.9%] female; 409 [0.4%] American Indian or Alaska Native; 2817 [2.4%] Asian; 76 725 [16.8%] Black; 11 131 [9.5%] Hispanic; 81 226 [69.0%] White; and 2373 [2.0%] other race or ethnicity; 27 078 [23.0%] dual-eligible; mean [SD] age, 71.2 [10.4] years). Following a contract termination, 20.1% (95% CI, 19.9%-20.4%) of enrollees switched to TM, including 32.7% (95% CI, 32.4%-33.1%) of dual-eligible beneficiaries and 16.4% (95% CI, 16.2%-16.5%) of non–dual-eligible beneficiaries. In nonterminated contracts, the concurrent switch rate was 6.2% (95% CI, 6.2%-6.2%) for all, 10.4% (95% CI, 10.4%-10.4%) for dual-eligible beneficiaries and 5.1% (95% CI, 5.1%-5.1%) for non–dual-eligible enrollees. The highest switch rates to TM were among Black enrollees (32.3% [95% CI, 31.7%-32.8%]) and those with prior use of hospital (31.3% [95% CI, 30.7%-31.9%], nursing home, 41.4% [95% CI, 40.4%-42.4%], or home health care (28.3% [95% CI, 27.4%-29.2%]). Beneficiaries who stayed in MA selected higher-rated star plans (mean posttermination contract star rating of 3.8 [95% CI, 3.8-3.8] stars compared with 3.3 [95% CI, 3.3-3.3] stars in the terminated year), but did not pay more in monthly premiums with 66.5% (95% CI, 66.2%-66.8%) paying the same or lower premiums.

**Conclusions and Relevance:**

In this cross-sectional study, 1 in 5 MA beneficiaries switched to TM after a contract termination, with Black beneficiaries and those with more intensive health needs having the highest switch rates. These findings highlight the need to examine consequences of contract terminations and subsequent insurance destinations on access to care and health outcomes, especially among those with marginalized race and ethnicity, those who are dual-eligible, and beneficiaries with higher health care needs.

## Key Points

**Question:**

After a Medicare Advantage (MA) contract termination, what insurance type do affected beneficiaries enroll in next?

**Findings:**

In this cross-sectional study of 117 681 MA beneficiaries who experienced contract termination from 2016 to 2018, 20.1% switched to traditional Medicare, with Black and dual-eligible beneficiaries having the highest switch rates. Beneficiaries who stayed in the MA program selected higher-rated star plans in the following year.

**Meaning:**

These results suggest that contract terminations are associated with high rates of exit from the MA program and, among those who remain in MA, enrollment in higher-rated plans; the potential impact of terminations on disparities in access to care and health outcomes deserves further investigation.

## Teaser

This cross-sectional study investigates insurance enrollment choices of Medicare Advantage beneficiaries whose contracts are terminated and assesses the characteristics of enrollees who switch to traditional Medicare after contract termination.

## Introduction

Enrollment in the Medicare Advantage (MA) program has increased substantially in recent years, now constituting 51% of the Medicare population.^1^ Moreover, recent growth in MA enrollment has been greater among Black, Hispanic, and low-income beneficiaries.^2^ Minoritized racial and ethnic beneficiaries had more MA plans available in their county of residence compared with White enrollees, but a higher proportion of these plans were low-rated.^3^ Plans vary in the benefits they offer, the quality of care they deliver, and the amount of cost sharing through premiums, copayments, and out-of-pocket payment maximums.^4^

Private companies choose to enter into contracts with the Centers for Medicare & Medicaid Services (CMS), where companies specify the benefits they will offer and estimate how much it will cost to offer those services. These contracts are submitted and renewed each year. Insurers have the option to terminate their MA contracts annually if it no longer is financially viable or not meeting their business needs. This may happen when an insurer wants to avoid providing a plan to a perceived risky beneficiary pool. In a separate process, CMS also has the authority to terminate an insurer’s contract in cases of sustained poor performance. Thus, MA contracts can have differing causes of termination. However, no matter the cause of the termination, these terminations may be of concern to beneficiaries as they can potentially disrupt their access to health care. When a beneficiary’s contract is terminated, they must transition to a different MA plan or to TM, which could entail substantial variations in benefits, out-of-pocket costs, physician networks, and prescription drug formularies.^5,6^ These plan decisions can be challenging due to the number of plans available and various information sources that beneficiaries use.^6,7^ The terminations may also affect Medicare beneficiary subgroups nonuniformly. For example, dual-eligible beneficiaries, those eligible for both Medicare and Medicaid, have unique health needs and greater health care spending,^8,9^ which may be especially impacted by changes in insurance plan or design.

Previous work found that approximately 1 in 5 MA contracts were terminated in the last decade, affecting more than 750 000 beneficiaries.^10^ We also found that these terminations may have had a disproportionate effect on Black beneficiaries who enrolled in terminated contracts at higher rates.^10^ This study examined the following 3 questions: (1) What are the insurance destinations of MA enrollees in the year immediately after contract termination? (2) What types of beneficiaries stay in MA or switch to TM following an MA termination? (3) Among those who remain in MA following a contract termination, to what extent do beneficiaries move to plans with higher measured quality or lower premiums?

## Methods

### Data Sources

Our primary source of data for this analysis was the Medicare Master Beneficiary Summary File (MBSF) spanning from January 1, 2016, to December 30, 2019. The MBSF contains individual records for each Medicare beneficiary for each year, including information on MA contract and plan enrollment, as well as beneficiary demographics. This cross-sectional study was determined to be exempt from review and granted a waiver of informed consent by the institutional review board at Brown University. This exemption was granted because the data used in the study were deidentified, and obtaining informed consent from a national sample was not a feasible option. The study adhered to the guidelines outlined in the Strengthening the Reporting of Observational Studies in Epidemiology (STROBE) reporting guideline.

In addition to utilizing the MBSF, we linked to publicly accessible files containing MA plan characteristics and star ratings, including a file that identified which plans were special needs plans. To evaluate beneficiary care utilization, we linked to the Medicare Provider Analysis and Review file for hospitalization data, the Outcome and Assessment Information Set for home health visits, and the Minimum Data Set (MDS) for nursing home stays which all report utilization for MA enrollees.

### Study Sample

The primary study sample included all Medicare beneficiaries with any MA enrollment who experienced a contract termination from January 1, 2016, through December 31, 2018. A comparison study sample included all Medicare beneficiaries with any MA enrollment during the same period and who resided in a county where a contract termination occurred. Of the 93 993 beneficiary-year observations among those who stayed in the MA program, we excluded 61 individual-years who were recorded to have died on January 1 of the subsequent year and 2949 individual-years with unknown plan information in the posttermination year in our analyses of changes in plan characteristics, leaving a sample size of 90 983 beneficiary-year observations for the analyses of those who stayed in MA.

### Plan Termination

Our primary independent variable was termination of a MA contract between one year and the next. We identified these terminations using the CMS plan crosswalk file, which classifies plans and contract IDs that were either terminated voluntarily by MA organizations, or involuntarily by CMS. Our data cannot distinguish between CMS vs plan-initiated terminations. However, CMS was prohibited by the 21st Century Cures Act from terminating contracts where there was sustained poor performance (3 consecutive years of a sub-3-star-rating) from 2016 to 2018,^11^ so it is very likely that the terminations of focus in this study were initiated by the insurer. Furthermore, both types of terminations have similar consequences for beneficiaries with respect to identifying a new option from the MA market or switching to TM.

### Outcomes

Our primary outcome was whether a beneficiary chose to switch to TM or to another plan following a termination. For both termination and nontermination groups, we also evaluated whether a beneficiary had access to another high-quality (rated 4 stars or higher) in their county. For those who remained in MA, we evaluated the plan-level characteristics of the contract they newly enrolled in, including plan types (HMO vs PPO), whether the plan was integrated with a health care delivery system, premiums, and star ratings. We excluded dual Medicaid beneficiaries for analyses of plan premiums because Medicaid pays their premiums. We determined which contracts were vertically integrated with health systems using a previously published approach.^12^

### Covariates

Our 2 primary patient-level variables of interest were dual-eligibility status and race and ethnicity as determined by the MBSF. Race and ethnicity was identified using the RTI race code available within the MBSF.^13^ The RTI code relies on race and ethnicity information sourced from Social Security information and refined through an algorithm to enhance the accurate reporting of race and ethnicity, especially improving identification of Hispanic ethnicity. Race and ethnicity were included in the analysis to identify socially disadvantaged groups in the US, and as proxies for exposure to systemic racism. Due to sample size, we limited the race and ethnicity analyses to Asian, Black, Hispanic, and White beneficiaries. Our other patient-level variables include age, sex, and use of hospital, nursing home, and home health care in the year before plan termination.

### Statistical Analysis

We first compared those beneficiaries who switched to TM with those who remained in the program posttermination by demographic and clinical characteristics. Plan information such as vertical integration, plan type, plan star rating, and premium were also included. We also compared the switch rate to TM for those in terminated contracts with the switch rate for the MA population that had not experienced termination but resided in a county where a plan termination had occurred.

Next, for those with terminated contracts who remained in MA, we examined changes in mean premiums and mean plan star ratings, stratified by race, ethnicity, and dual status. We compared characteristics using χ^2^ and *t* tests. A statistical significance threshold of *P* < .05 with 2-tailed tests was used. Data analysis took place from June 2023 to April 2024. All analyses were conducted in Stata version 17 (StataCorp LLC).

## Results

The primary sample included 117 681 beneficiary-year observations, of which a total of 64 654 (54.9%) were female and 53 027 (45.1%) were male; 409 (0.4%) were American Indian or Alaska Native, 2817 (2.4%) were Asian, 76 725 (16.8%) were Black, 11 131 (9.5%) were Hispanic, 81 226 (69.0%) were White, and 2373 (2.0%) were other race or ethnicity; 27 078 (23.0%) were dual-eligible; and the mean (SD) age was 71.2 (10.4) years. From the primary sample, 93 993 (79.9%) remained in the MA program after experiencing contract termination and 23 688 (20.1%; 95% CI, 19.9%-20.4%) switched to TM. The Table depicts the demographic, plan information, and utilization of both groups stratified by dual-eligibility status. Compared with those remaining in MA, the group that switched to TM was younger (mean [SD] age, 69.7 [12.8] vs 71.6 [9.7] years; 95% CI of difference: −2.1 to −1.8 years; *P* < .001), had a higher proportion who were Black (6360 [26.9%] vs 13 365 [14.2%]) or Hispanic (3106 [13.1%] vs 8025 [8.5%]), more likely to be in terminated plans that were terminated HMOs (19 030 [80.3%] vs 63 283 [67.3%]), were vertically integrated (10 813 [45.7%] vs 50 967 [56.6%]), had slightly lower monthly premiums for non–dual-eligible beneficiaries (mean [SD] premium: $39.31 [27.15] vs $39.97 [28.72]; 95% CI of difference: −$1.16 to −$0.15], and a lower star rating (mean [SD] plan star rating: 3.1 [0.4] vs 3.3 [0.4] stars; 95% CI of difference: −0.17 to −0.16 stars).

**Table.** Demographics, Plan Characteristics, and Utilization of Beneficiaries by Posttermination Insurance and Dual-Eligibility Status

Variables	Overall			Non–dual-eligible			Dual-eligible		
	Remained in MA (n = 93 993)	Switched to TM (n = 23 688)	*P* value	Remained in MA (n = 75 780)	Switched to TM (n = 14 823)	*P* value	Remained in MA (n = 18 213)	Switched to TM (n = 8865)	*P* value
Age, mean (SD), y	71.6 (9.7)	69.7 (12.8)	<.001	72.6 (8.60)	72.0 (10.4)	<.001	67.5 (12.6)	65.9 (15.2)	<.001
Sex, No. (%)									
Female	52 185 (55.5)	12 469 (52.6)	<.001	41 252 (54.4)	7417 (50.0)	<.001	10 933 (60.0)	5052 (57.0)	<.001
Male	41 808 (45.5)	11 219 (47.4)	<.001	34 528 (45.6)	7406 (50.0)	<.001	7280 (40.0)	3813 (43.0)	<.001
Race and ethnicity, No. (%)									
American Indian or Alaska Native	279 (0.3)	130 (0.6)	<.001	189 (0.3)	70 (0.5)	<.001	90 (0.5)	60 (0.7)	<.001
Asian	2287 (2.4)	530 (2.2)		1413 (1.9)	231 (1.6)		474 (4.8)	299 (3.4)	
Black	13 365 (14.2)	6360 (26.9)		8983 (11.9)	3417 (23.1)		4382 (24.1)	2943 (33.2)	
Hispanic	8025 (8.5)	3106 (13.1)		3609 (4.8)	1229 (8.3)		4416 (24.3)	1877 (21.2)	
Other^a^ or unknown	1897 (2.0)	476 (2.0)		1530 (2.0)	323 (2.2)		367 (2.0)	153 (1.7)	
White	68 140 (72.50)	13 086 (55.2)		60 656 (79.3)	9553 (64.5)		8084 (44.4)	3533 (39.9)	
Plan type, No. (%)									
HMO	61 244 (67.3)	19 030 (80.34)	<.001	46 935 (63.1)	11 005 (74.2)	<.001	14 309 (86.5)	8025 (90.5)	<.001
PPO	29 739 (32.7)	4658 (19.66)		27 503 (36.9)	3818 (25.8)		2236 (13.5)	840 (9.5)	
Vertical integration, No. (%)	52 005 (57.2)	10 813 (45.7)	<.001	45 916 (61.7)	7106 (47.9)	<.001	6089 (36.8)	3703 (41.8)	<.001
Premium, mean (SD), $	32.13 (27.37)	36.42 (24.26)	<.001	39.97 (28.72)	39.31 (27.15)	.0053	NA	NA	NA
Zero premium, No. (%)	15 969 (17.0)	2587 (10.9)	<.001	14 239 (18.8)	2114 (14.3)	<.001	NA	NA	NA
Star level, mean (SD)	3.3 (0.43)	3.1 (0.39)	<.001	3.4 (0.41)	3.2 (0.39)	<.001	3.1 (0.44)	3.0 (0.36)	<.001
Star level, No. (%)									
2-2.5	4576 (5.0)	2394 (10.1)	<.001	1587 (2.1)	1064 (7.2)	<.001	2989 (18.1)	1330 (15.0)	<.001
3-3.5	62 975 (69.2)	11 318 (47.8)		52 702 (70.8)	10 508 (70.8)		10 273 (62.1)	5687 (641.2)	
4-4.5	14 129 (15.5)	4877 (20.6)		12 832 (17.2)	1371 (9.3)		1297 (7.8)	259 (2.9)	
5	0	0		0	0		0	0	
Plan too new or not enough data	9303 (10.2)	3469 (14.6)		7317 (9.8)	1880 (12.7)		1986 (12.0)	1589 (17.9)	
Utilization, No. (%)									
Hospitalization	11 941 (12.7)	5439 (23.0)	<.001	8665 (11.4)	3157 (21.3)	<.001	3276 (18.0)	2282 (25.7)	<.001
Nursing home	3281 (3.5)	2317 (9.8)	<.001	2107 (2.8)	1101 (7.4)	<.001	1174 (6.5)	1216 (13.7)	<.001
Home health	5955 (6.3)	2350 (9.9)	<.001	418 (5.5)	1355 (9.1)	<.001	1787 (9.8)	995 (11.2)	<.001

Abbreviations: HMO, Health Maintenance Organization; MA, Medicare Advantage; NA, not applicable; PPO, preferred provider organization; TM, traditional Medicare.

^a^ In the race and ethnicity identification (RTI) codes, the other category typically refers to individuals who do not identify with the specific racial or ethnic groups listed. This can include people from mixed backgrounds or those who identify with a racial or ethnic group not explicitly mentioned in the available categories.

**Figure 1.**
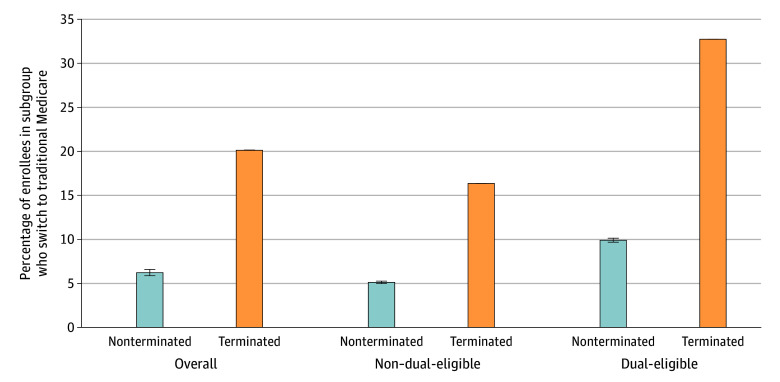
Percentage Switch Rate to Traditional Medicare by Dual-Eligibility Status and Contract Termination, 2016 to 2019 The figure shows the percentage of beneficiaries in a group who switch to traditional Medicare. The groups are separated by whether a beneficiary experienced a contract termination and whether they are dual-eligible. The whiskers indicate 95% CIs.

Figure 1 shows the switch rate to TM by beneficiaries in terminated and nonterminated plans stratified by dual status. Overall, 23 688 beneficiaries (20.1% [95% CI, 19.9%-20.4%]) in terminated plans switched to TM compared with 1.57 million (6.2% [95% CI, 6.2%-6.2%]) of their 24.3 million counterparts in nonterminated plans. When looking specifically at the non–dual-eligible population, 994 031 (5.1% [95% CI, 5.1%-5.1%]) of beneficiaries in nonterminated plans switched to TM compared with 14 823 (16.4% [95% CI, 16.2%-16.5%]) of beneficiaries in terminated plans. In the dual-eligible population, 8865 (32.7% [95% CI, 32.4%-33.1%) of dual-eligible beneficiaries in terminated plans switched to TM, whereas 578 645 (9.9% [95% CI, 9.9%-9.9%]) of dual-eligible beneficiaries in nonterminated plans. In our study population, 19 129 dual-eligible beneficiaries (70.6%) were in special needs plans (SNPs) prior to termination. Of these, 2816 (14.7%) switched to a non-SNP post termination, 9442 (49.4%) switched to another SNP, and 6871 (35.9%) switched to TM. In the nonterminated population over the same timeframe, 4 231 119 (72.5%) of dual-eligible beneficiaries were in SNPs, of which 100 282 (2.6%) switched to a non-SNP the following year and 366 886 (8.7%) switched to TM. Of the 117 681 beneficiaries who experienced contract termination, 6503 (5.5%) did not have access to a high-rated plan in their county, and 1546 (23.8%) of these individuals switched to TM compared with 21 918 (19.9%) who had access to a high-rated plan in their county. In our comparison group of 24.3 million beneficiaries in nonterminated plans, 89 554 (0.4%) did not have access to a high-rated plan in their county, and 10 975 (12.3%) of these individuals switched to TM compared with 1.56 million (6.5%) who had access to a high-rated plan in their county.

**Figure 2.**
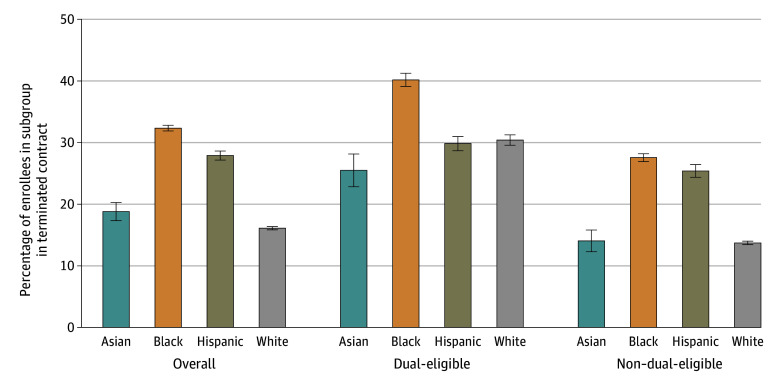
Percentage Who Switch to Traditional Medicare When in a Terminated Contract by Race and Ethnicity and Dual-Eligibility Status, 2016 to 2019 The figure shows the percentage of beneficiaries from each race and ethnicity who switched to traditional Medicare after experiencing contract termination. Sample sizes precluded analyses of American Indian or Alaska Native and other race and ethnicity groups. The whiskers indicate 95% CIs.

Figure 2 illustrates the percentage of enrollees in terminated plans who switched to TM by race and ethnicity and dual-eligibility status. Black beneficiaries had the highest switch rates to TM at 32.3% (95% CI, 31.7%-32.8%), including 40.2% (95% CI, 39.1%-41.3%) for dual-eligible, and 27.6% (95% CI, 26.9%-28.2%) for non–dual-eligible. Generally, Asian and White non–dual-eligible beneficiaries had the lowest switch rates at 14.1% (95% CI, 12.3%-15.8%) and 13.7% (95% CI, 13.5%-14.0%), respectively.

**Figure 3.**
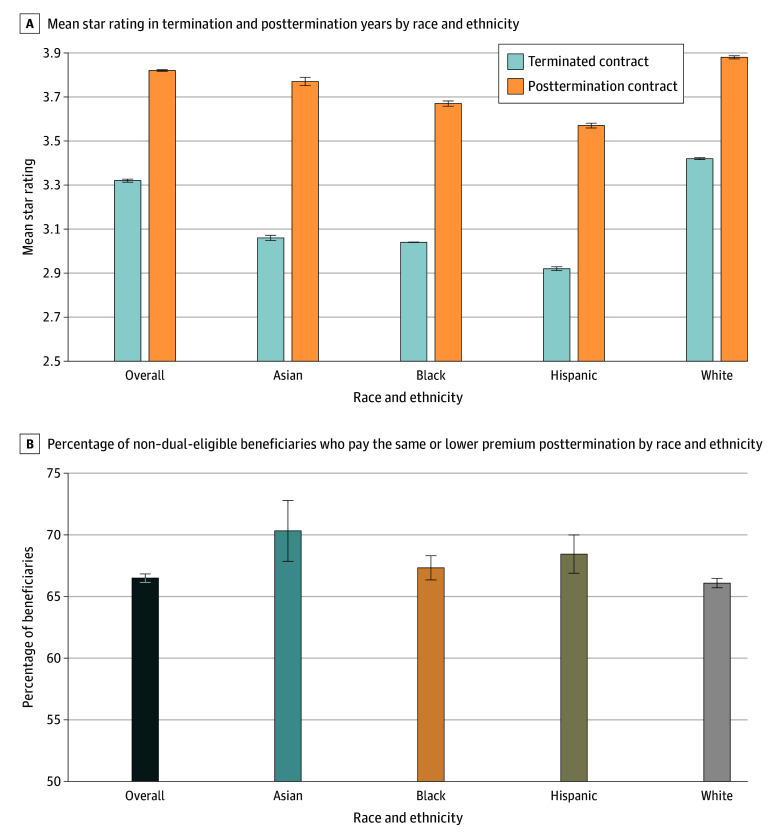
Mean Star Rating in Termination and Posttermination Years by Race and Ethnicity A, Mean star rating in termination and posttermination years by race and ethnicity: the figure depicts the mean plan star rating of a beneficiary's Medicare Advantage plan in the year their plan was terminated and the subsequent year. The mean star rating in termination and posttermination years are also shown by race and ethnicity. This comparison is limited to the 90 983 of 93 993 beneficiaries who (1) stayed in Medicare Advantage after contract termination, (2) survived past January 1 of the following year, and (3) for whom the following year's plan rating was available. B, Percentage of non–dual-eligible beneficiaries who pay the same or lower premium posttermination by race and ethnicity: this figure shows the percentage of non–dual-eligible beneficiaries by racial group who either pay the same or lower monthly premium the year following a contract termination. The whiskers indicate 95% CIs.

Figure 3A shows the mean plan star rating of the terminated and posttermination contracts stratified by race and ethnicity. The mean star rating of a terminated contract was 3.3 (95% CI, 3.3-3.3) stars, whereas the mean posttermination contract star rating was 3.8 (95% CI, 3.8-3.8) stars. It is important to note, however, that 27 of the 52 terminated plans (51.9%) from 2016 to 2018 were deemed by CMS to be too new or not have enough data to give a corresponding star rating. Although each racial and ethnic group experienced an increase in mean plan star rating post termination, the biggest increases in mean star rating were observed among Asian (3.1 [95% CI, 3.0-3.1] stars to 3.8 [95% CI, 3.7-3.8] stars) and Hispanic (2.9 [95% CI, 2.9-2.9] stars to 3.6 [95% CI, 3.6-3.6] stars) enrollees. Notably, all minoritized racial and ethnic beneficiaries had lower mean plan star ratings in both the terminated and posttermination contracts compared with White beneficiaries (3.4 [95% CI, 3.4-3.4] stars to 3.9 [95% CI, 3.9-3.9] stars).

Figure 3B compares the percentage of beneficiaries in terminated plans that paid the same or lower premiums the year after termination by race and ethnicity. The majority (66.5% [95% CI, 66.2%-66.8%) of non–dual-eligible beneficiaries across racial and ethnic groups did not pay higher monthly premiums. Asian beneficiaries had the highest proportion (70.3% [95% CI, 67.9%-72.8%]) of individuals, whereas White beneficiaries had the lowest (66.1% [95% CI, 65.7%-66.5%]), who paid the same or a lower premium in the year post termination.

**Figure 4.**
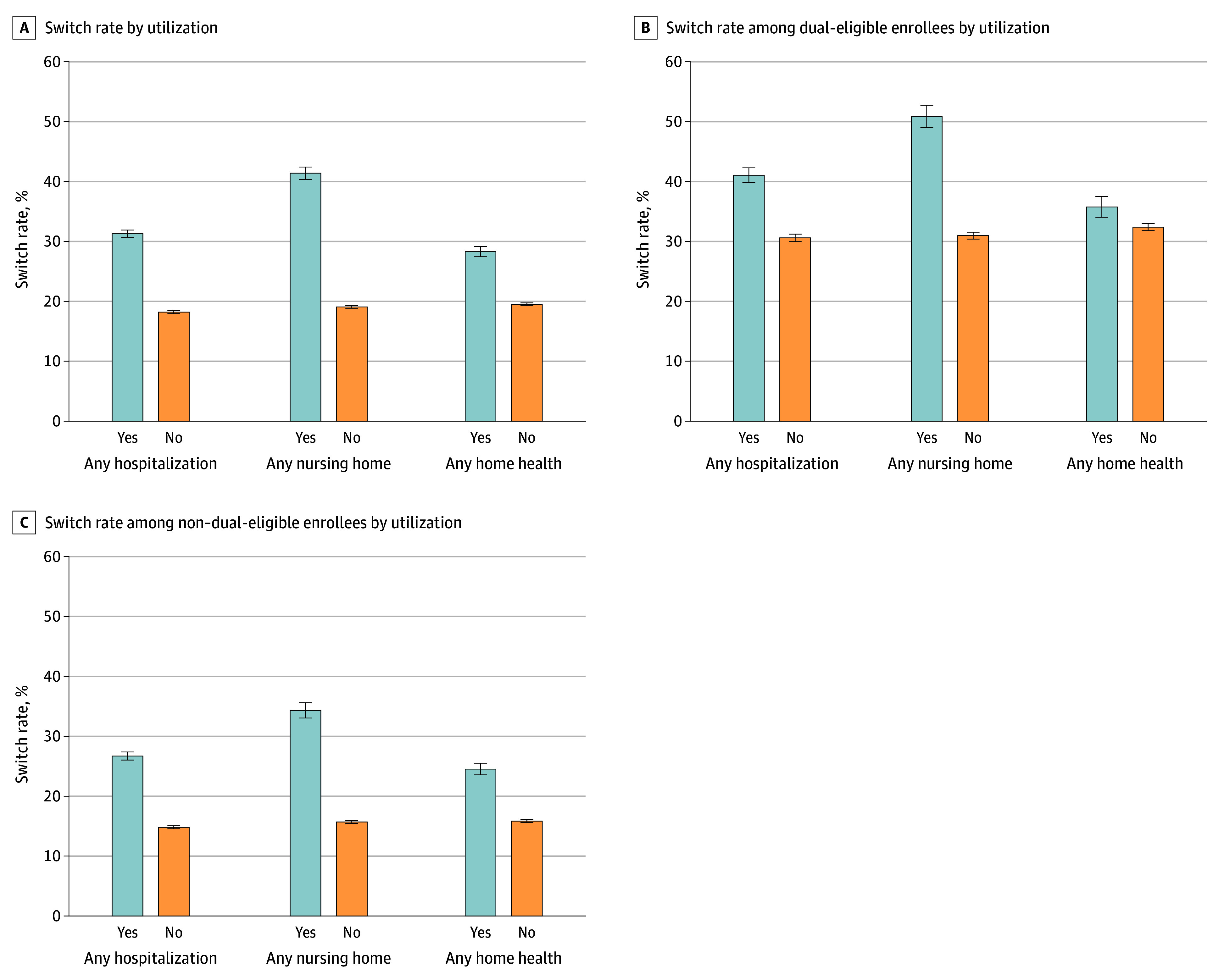
Switch Rate to Traditional Medicare by Utilization A, Switch rate to traditional Medicare by utilization: the figure depicts the percentage of beneficiaries who switched to traditional Medicare if they utilized acute or postacute care the year their plan was terminated. B, Switch rate among dual-eligible beneficiaries by utilization: this figure shows the percentage of dual-eligible beneficiaries who switched to traditional Medicare if they utilized acute or postacute care the year their plan was terminated. C, Switch rate among non–dual-eligible beneficiaries by utilization: this figure shows the percentage of non–dual-eligible beneficiaries who switch to traditional Medicare if they utilized acute or postacute care the year their plan was terminated. The whiskers indicate 95% CIs.

Figure 4A compares the beneficiary switch rate to TM by any (at least 1 during the year) hospital, nursing home, or home health utilization in the year prior to termination. Panels B and C of Figure 4 make the same comparisons among dual-eligible and non–dual-eligible beneficiaries, respectively. Across overall, dual-eligible, and non–dual-eligible strata, those who used hospital, nursing home, or home health care consistently had a greater switch rate to TM compared with beneficiaries who did not use these services.

### Sensitivity Analysis

eTable 1 in Supplement 1 shows the racial and ethnic and dual-eligible and non–dual-eligible characteristics of beneficiaries in our sample who switched to TM, and eTable 2 in Supplement 1 shows the same characteristics for the beneficiaries in nonterminated plans in counties where a contract termination occurred over the same period who switched to TM. eTable 3 in Supplement 1 compares the plan characteristics (plan type, premium, star level, and any vertical integration) for the terminated population that stayed in MA, the terminated population that switched to TM, and the posttermination population that stayed in MA (90 983 beneficiaries). eTable 4 in Supplement 1 shows that 57 661 (63.4%) stayed in the plan type they were originally enrolled in, 58 129 (63.9%) paid the same or lower premium, 49 912 (54.9%) switched to a higher-rated plan, and 76 445 (84.0%) switched to plans without vertical integration (or were never in plans with any vertical integration). eTable 5 in Supplement 1 shows that high proportions of terminated MA plans were vertically integrated.

## Discussion

Our study has 4 key findings. First, 20.1% of beneficiaries, including 16.4% of non–dual-eligible and 32.74% of dual-eligible beneficiaries, who experienced MA contract termination switched to TM in the next year. Second, contract termination resulted in more than 50% of dual-eligible beneficiaries in SNPs moving to a non-SNP or switching to TM. Third, Black beneficiaries in all overall, dual-eligible, and non–dual-eligible groups and those with prior use of hospital, nursing home, or home health care had the highest switch rates to TM. Lastly, beneficiaries who stayed in the MA program selected higher-rated star plans in the following year but did not pay more in monthly premiums.

Our findings suggest that in some ways contract termination is working as intended to remove low-rated plans from the MA program as beneficiaries tended to enroll in higher-quality plans. This could be a function of CMS terminating poor-performing contracts, but this is unlikely because CMS was prohibited from terminating contracts for repeated poor performance during our study period by the 21st Century Cures Act. On the other hand, MA insurers may be strategically terminating contracts with beneficiaries whom they perceive as risky and pass on that risk to TM or other plans. These mechanisms are not necessarily mutually exclusive, but the end result is the same: disruption to a patient’s care experience. While prior research has found that the termination of lower-quality contracts may be beneficial in reducing mortality in some enrollees,^14^ we find substantial variation in the quality of destination contracts by subgroup. Furthermore, terminations lead to more beneficiaries enrolling in TM, which may expose those who are unable to afford or enroll in Medigap coverage to face substantially higher health care costs. This is of particular concern as in most states, enrollees may be denied Medigap coverage on the basis of preexisting conditions if they were previously enrolled in an MA plan.^15^

Because MA plans often contract with a limited set of clinicians in their network designs, switching from one MA plan to another may result in a beneficiary experiencing disruption in access to care because their clinician is no longer in-network. Given the potential disproportionate effect of contract terminations on Black beneficiaries^10^ and this study’s findings of elevated switch rates to TM among the same group, Black beneficiaries may be especially at risk for care disruptions or unanticipated expenses as a result of contract termination.

### Limitations

Our study has limitations. First, this analysis was associational and cannot evaluate causality. Next, we were not able to identify whether plans terminated the contract themselves or whether the event was CMS’s decision, but in either case there is a potential disruption to an enrollee’s continuity of coverage. Based on the study period, it is very likely that the plans terminated the contract themselves because CMS did not have the authority to terminate contracts based on poor performance during most of the study period and we did not identify any terminated contracts with less than 3 stars. Similarly, our data did not provide information about whether beneficiaries chose to switch to TM or were defaulted to that decision because they did not select a new MA plan.

## Conclusions

In this cross-sectional study, we found that 16.4% of non–dual-eligible and 32.7% of dual-eligible MA beneficiaries in terminated contracts switched to TM, with variation by race and ethnicity. Of those who stayed in MA, we found that most of these individuals switched to higher-rated star plans but did not typically pay more in monthly premiums as a result. As the MA program continues to grow and plan terminations continue, more attention may be needed to ensure that beneficiaries have a smooth transition to their subsequent insurer after experiencing contract termination and do not experience a disruption in care.

## Notes

Supplement 1. **eTable 1.** Beneficiaries in Terminated Plans Who Switch to TM **eTable 2.** Beneficiaries in Non-Terminated Plans Who Switch to TM **eTable 3.** Plan Characteristics of Beneficiaries Who Experience Contract Termination **eTable 4.** Change in Beneficiary’s Plan Characteristics for those Who Experienced Termination **eTable 5.** Terminated MA Plans With Any Vertical Integration From 2016-2019

Supplement 2.
Data Sharing Statement
