# Supplementary material for: Contract Termination and Insurance Enrollment Among Medicare Advantage Beneficiaries
Source: JAMA Netw Open. 2024 Aug 20;7(8):e2428267. doi: 10.1001/jamanetworkopen.2024.28267 (PMC11337069; doi:10.1001/jamanetworkopen.2024.28267)
Supplement: Supplement 1. — eTable 1. Beneficiaries in Terminated Plans Who Switch to TM eTable 2. Beneficiaries in Non-Terminated Plans Who Switch to TM eTable 3. Plan Characteristics of Beneficiaries Who Experience Contract Termination eTable 4. Change in Beneficiary’s Plan Characteristics for those Who Experienced Termination eTable 5. Terminated MA Plans With Any Vertical Integration From 2016-2019 [file jamanetwopen-e2428267-s001.pdf]

Supplemental Online Content

Dixit MN, Trivedi AN, Meyers DJ. Contract termination and insurance enrollment among Medicare Advantage beneficiaries. *JAMA Netw Open.* 2024;7(8):e2428267. doi:10.1001/jamanetworkopen.2024.28267

eTable 1. Beneficiaries in Terminated Plans Who Switch to TM

eTable 2. Beneficiaries in Non-Terminated Plans Who Switch to TM

eTable 3. Plan Characteristics of Beneficiaries Who Experience Contract Termination

eTable 4. Change in Beneficiary's Plan Characteristics for Those Who Experienced Termination

eTable 5. Terminated MA Plans With Any Vertical Integration From 2016-2019

This supplemental material has been provided by the authors to give readers additional information about their work.

© 2024 Dixit MN et al. *JAMA Network Open*.

eTable 1: Beneficiaries in Terminated Plans who Switch to TM

n (%)	Dual	Non-Dual	Total
Asian	299 (25.49)	231 (14.05)	530 (18.81)
Black	2943 (40.18)	3417 (27.56)	6360 (32.34)
Hispanic	1877 (29.83)	1229 (25.40)	3106 (27.9)
NA/AN	60 (40.00)	70 (27.03)	130 (31.78)
Other/Unknown	153 (29.42)	323 (17.43)	476 (20.06)
White	3533 (30.41)	9553 (13.72)	13086 (16.11)
Total	8865 (32.74)	14823 (16.36)	23688

Note: Percentages are calculated as the proportion of people from a dual/racial group who experience a termination that switch to Traditional Medicare

eTable 2: Beneficiaries in Non-Terminated Plans Who Switch to TM

n (%)	Dual	Non-Dual	Total
Asian	48,131 (8.5)	36,398 (3.9)	84,529 (5.6)
Black	133,283 (10.2)	110,700 (5.2)	243,983 (7.1)
Hispanic	137,069 (7.51)	94,320 (4.02)	231,389 (5.6)
NA/AN	8,614 (9.2)	17,724 (3.8)	26,338 (4.7)
Other/Unknown	1,989 (11.4)	1,824 (6.6)	3,823 (8.5)
White	249,559 (12.3)	733,055 (5.4)	982,614 (6.3)
Total	578,645 (9.9)	994,031 (5.1)	1,572,676

Note: Percentages are calculated as the proportion of people from a dual/racial group who do not experience a contract termination but reside in the same county where a termination occurred that switch to Traditional Medicare.

© 2024 Dixit MN et al. *JAMA Network Open*.

eTable 3: Plan Characteristics of Beneficiaries who Experience Contract Termination

		Terminated Contract and Stay in MA	Terminated Contract but Switch to TM	Post-Termination Contract (Stay in MA)
Plan Type (n, %)	HMO	61244 (67.3)	19030 (80.34)	56236 (61.8)
	PPO	29739 (32.7)	4658 (19.66)	34747 (38.2)
	Total	90983	23688	90983
Vertical Integration	(n, %)	52005 (57.16)	10813 (45.65)	14538 (15.98)
Premium	Mean (SD)	32.13 (0.1208088)	36.42272 (0.1576152)	33.72 (0.1298)
Zero Premium	n (%)	34761 (38.9)	2587 (10.92)	33429 (37.2)
	Total	89355	23688	89966
Star Level	Mean (SD)	3.3152 (0.0014543)	3.14202 (0.002727)	3.821082 (0.0016451)
n (%)	2-2.5	4576 (5.03)	2394 (10.11)	2218 (2.44)
	3-3.5	62975 (69.22)	11318 (47.78)	34756 (38.2)
	4-4.5	14129 (15.53)	4877 (20.59)	48407 (53.2)
	5	0	0	703 (0.77)
	Plan too new / not enough data	9303 (10.22)	3469 (14.64)	4899 (5.38)
	Total	90983	23688	90983

© 2024 Dixit MN et al. *JAMA Network Open*.

eTable 4: Change in Beneficiary's Plan Characteristics for those Who Experienced Termination

Change in Plan Type (n,%)	stay HMO	42079 (46.25)
	stay PPO	15582 (17.13)
	HMO to PPO	19165 (21.06)
	PPO to HMO	14157 (15.56)
Change in Premium, n (%)	same premium	27534 (30.26)
	higher premium	32854 (36.11)
	lower premium	30595 (33.63)
Change in Zero Premium, n (%)	stay zero	25151 (27.64)
	switch to zero	8278 (9.10)
	switch from zero	9610 (10.56)
	never zero	47944 (52.70)
Change in Star Level, n (%)	same star	26060 (28.64)
	higher star	49912 (54.86)
	lower star	1841 (2.02)
	unknown	13170 (14.48)
Change in Plan Vertical Integration, n (%)	stay no int	36771 (40.42)
	stay int	12331 (13.55)
	switch to int	2207 (2.43)
	switch to no int	39674 (43.61)

© 2024 Dixit MN et al. *JAMA Network Open*.

eTable 5: Terminated MA Plans with any Vertical Integration from 2016-2019

Year	MA Plans with any Vertical Integration	Terminated MA Plans	Terminated MA Plans with any Vertical Integration, n (%)
2016	104	21	10 (47.6)
2017	95	14	0 (0)
2018	103	17	8 (47.1)
2019	104	8	2 (25.0)
total	424	50	21

© 2024 Dixit MN et al. *JAMA Network Open*.
