# Supplementary material for: Contract Termination and Insurance Enrollment Among Medicare Advantage Beneficiaries
Source: JAMA Netw Open. 2024 Aug 20;7(8):e2428267. doi: 10.1001/jamanetworkopen.2024.28267 (PMC11337069; doi:10.1001/jamanetworkopen.2024.28267)
Supplement: Supplement 2. — Data Sharing Statement [file jamanetwopen-e2428267-s002.pdf]

Data Sharing Statement

Dixit. Contract Termination and Insurance Enrollment Among Medicare Advantage Beneficiaries. *JAMA Netw Open*. Published August 20, 2024.
doi:10.1001/jamanetworkopen.2024.28267

Data
Data available: No

Additional Information
Explanation for why data not available: We can't share raw data as it is covered by a DUA with CMS. Code can be made available upon request.
